# Utility of an Externalized Temporary Transvenous Implantable Cardioverter-defibrillator System in the Setting of Ventricular Tachycardia Storm and Concurrent Device Infection Requiring Extraction

**DOI:** 10.19102/icrm.2024.15071

**Published:** 2024-07-15

**Authors:** Ronuk M. Modi, Marianna Lozano Cruz Marquez, Shu Yang, Robert N. D’Angelo, Timothy R. Maher, Bahij Kreidieh, Nicholas O. Palmeri, Hans F. Stabenau, Dana Goldense, Emily Wacks, Patricia Tung, Andre d’Avila, Jonathan Waks, Peter Zimetbaum, Andrew H. Locke

**Affiliations:** 1 Division of Cardiology, Beth Israel Deaconess Medical Center, Boston, MA, USA; 2 Pontificia Universidade Catolica de Sao Paolo, Sao Paolo, Brazil; 3 South Denver Cardiology, Littleton, CO, USA

**Keywords:** Anti-tachycardia pacing, cardiac implantable electronic device infection, externalized pulse generator, implantable cardioverter-defibrillator, monomorphic ventricular tachycardia

## Abstract

With the expanding use of cardiac implantable electronic device (CIED) therapy, intravascular device infections are becoming more common. In the case of transvenous implantable cardioverter-defibrillator (ICD) infections requiring extraction for bacterial clearance, there remains no standard method to deliver temporary ICD therapy following device removal. We present a case of persistent bacteremia complicated by monomorphic ventricular tachycardia (VT) electrical storm where biventricular ICD system extraction was performed and a temporary transvenous dual-coil lead with an externalized ICD generator was used to treat VT episodes prior to the re-implantation of a new permanent system. This case demonstrates the utility of a temporary externalized transvenous ICD system in the successful detection and pace-termination of VT, thereby reducing episodes of painful and potentially harmful external defibrillator shocks during the treatment of CIED infection.

## Introduction

Cardiac implantable electronic device (CIED) infection is associated with substantial morbidity, mortality, and health care costs.^1–4^ The expanded use of CIEDs over recent years has led to an increase in the number of CIED infections. Optimal treatment strategies remain an area of evolving research interest.^5^ In particular, infective endocarditis associated with CIED (CIED-IE) is associated with increased short- and long-term mortality when compared to isolated CIED pocket infections.^2,3^ In cases of clear device involvement with high-grade bacteremia or high-risk organisms, prompt device extraction is indicated and is associated with improved outcomes.^1,3,4^ For pacemaker-dependent patients, the placement of an externalized pulse generator with an active fixation pacing lead is accepted as a strategy while awaiting re-implantation. However, similar strategies have not been widely described in patients with implantable cardioverter-defibrillators (ICDs) who require concurrent device therapies for ventricular tachycardia (VT). Here, we describe the use of an externalized ICD generator and a temporary transvenous dual-coil defibrillator lead in a patient with concurrent VT storm and CIED-IE.

## Case presentation

A 78-year-old man presented with dyspnea, hypotension, and VT storm. His past medical history included non-ischemic dilated cardiomyopathy, chronic heart failure with reduced ejection fraction, and recurrent monomorphic VT. He underwent initial implantation of a right-sided single-chamber dual-coil ICD in 2007 for primary prevention. In the setting of worsening left ventricular (LV) ejection fraction (15%) and left bundle branch block, he underwent a cardiac resynchronization therapy defibrillator (CRT-D) upgrade in 2016. His LV ejection fraction subsequently improved to 45% with New York Heart Association class I symptoms. In April 2022, due to an increasing burden of monomorphic VT requiring multiple episodes of anti-tachycardia pacing (ATP) and one ICD shock, he underwent VT ablation. During the procedure, clinical premature ventricular complexes and non-sustained VT were mapped to the LV summit and ablated in the distal coronary sinus and endocardially via a transseptal and retrograde approach. VT was not inducible with ventricular stimulation at the conclusion of the case. However, from the time of the ablation to the current presentation, he had required multiple further device therapies for VT. There were a total of 34 treated VT episodes over the 6-month period. One episode was accelerated by ATP and required a single device shock for termination, while the others were all terminated effectively by ATP.

**Figure 1:**
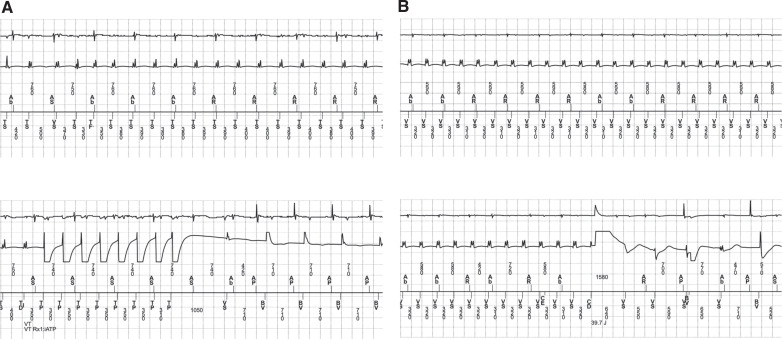
**A:** Intracardiac electrogram recordings displaying monomorphic ventricular tachycardia occurring during the described hospitalization with a cycle length of about 400 ms terminated successfully with anti-tachycardia pacing. **B:** Faster monomorphic ventricular tachycardia with a cycle length of about 320 ms occurring during the described hospitalization terminated with an implantable cardioverter-defibrillator shock.

On this presentation, he was initially admitted to a local community hospital with dyspnea and heart failure exacerbation. He was later transferred to our institution once he was found to have *Enterococcus faecalis* bacteremia. Blood cultures remained positive despite antibiotic therapy for several days, raising concern for CIED infection. Transesophageal echocardiography demonstrated an 0.8-cm filamentous mobile echodensity on the right atrial side of the tricuspid valve most consistent with a vegetation. No lead-associated vegetations were present. During this same hospitalization, the patient had five episodes of hemodynamically stable monomorphic VT leading to palpitations in a 6-h period despite ongoing amiodarone therapy. Four episodes of VT (cycle length [CL], 370–400 ms) were successfully treated with a single round of ATP, and one episode was accelerated after ATP (CL, 320 ms) and then successfully terminated with a single ICD shock **(Figure 1)**.

**Figure 2:**
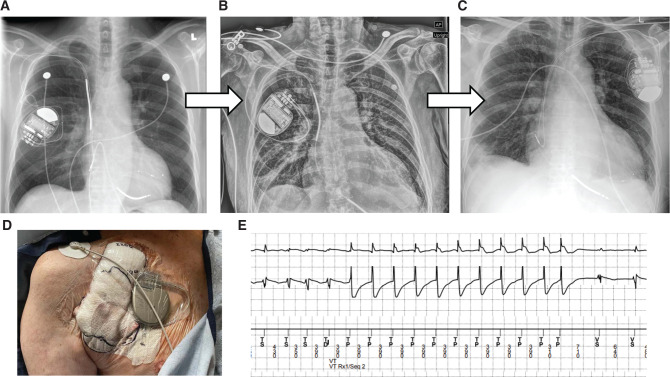
**A:** Admission chest X-ray showing the original right-sided cardiac resynchronization therapy (CRT-D) defibrillator dual-coil system. **B and D:** Temporary externalized single-chamber implantable cardioverter-defibrillator (ICD) system with a dual-coil DF1 RV ICD lead. **C**: Final left-sided CRT-D transvenous system following re-implantation 7 days after device extraction. **E**: Anti-tachycardia pacing therapy delivered by an externalized temporary ICD system successfully terminating ventricular tachycardia.

Given the evidence of CIED-IE, laser-assisted device extraction was performed to facilitate bacterial clearance. All three leads were successfully removed in their entirety without any retained fragments. No temporary pacing was needed as the patient had intact native atrioventricular conduction. However, given the frequent device therapies for VT, as mentioned already, a temporary transvenous right ventricular (RV) dual-coil DF1 lead (Sprint Quattro Secure 6947-65; Medtronic Inc., Minneapolis, MN, USA) was concurrently placed via the right axillary vein and connected to the original ICD generator in an externalized position at the right upper chest **(Figure 2)**. Programming was set to bipolar sensing and pacing. For ATP delivery and defibrillation therapy, a vector involving only the RV and superior vena cava (SVC) coils was used. All therapy vectors involving the externalized ICD generator were deactivated. The patient was monitored in a telemetry cardiac step-down unit. Over the subsequent days, the patient had multiple successful ATP therapies using the externalized ICD system without the need for device shocks or external defibrillation. Seven days later, with blood cultures remaining negative on antibiotic therapy, he underwent successful re-implantation of a new CRT-D system with left-sided generator placement **(Figure 2)**. He was successfully discharged with a plan to complete a 6-week course of antibiotic therapy.

Institutional patient consent was obtained for the use of patient images in medical publications, and this work was deemed to not require institutional review board approval.

## Discussion

The management of CIED infection requiring ICD extraction in the setting of concurrent ventricular arrhythmias requiring frequent device therapies is not well established. There are, however, several advantages to the approach described here when compared to alternative strategies, such as attempting conservative management with antibiotic suppression and device retention or explanting the device and relying on external defibrillation for the treatment of VT.

From the standpoint of CIED infection treatment, retaining a transvenous ICD to preserve the option for ongoing device-based VT therapy is less preferable to complete device removal. The treatment of CIED infection in patients who do not undergo complete hardware removal is associated with poorer outcomes and prognosis.^3,4,6^ The short-term mortality of CIED infection is estimated to be about 4%–8%, with greater rates observed in those with endocarditis as compared to those with pocket infections.^2,3^ In one retrospective study of CIED infections, treatment with antibiotic therapy alone without device removal was associated with a sevenfold increase in 30-day mortality. Immediate device removal was associated with a threefold decrease in 1-year mortality compared to both no device removal and delayed device removal.^7^ Another case series describing outcomes in patients who could not undergo device removal and were prescribed chronic suppressive antibiotics reported a 30-day mortality of 25%.^8^ Though this must be weighed against the risk of procedural complications and mortality with lead extraction, reported to occur in around 2%–3% and 0.5% of total cases, respectively, the balance is overall in favor of device removal when feasible. Both the Heart Rhythm Society and the European Heart Rhythm Association guidelines provide class I recommendations for device extraction in the setting of systemic CIED infection.^1,3^

In situations where ICD extraction is necessary for the treatment of infection, patients suffering from ongoing VT are left unprotected following extraction. In our experience, the risks of retaining the original system in the setting of CIED infection seem to far outweigh the benefits of treating possible recurrent VT. However, this patient had demonstrated recurrent monomorphic VT that was responsive to ATP, with several episodes in the months leading up to this hospitalization followed by an acute increase in VT burden in the setting of decompensated heart failure and infection. We suspected that he would likely have ongoing VT after device removal and did not want to subject him to frequent defibrillation via an external or wearable defibrillator. While secondary prevention defibrillators are a cornerstone of management in patients with known recurrent monomorphic VT, evidence suggests that ATP is a preferable treatment to ICD shocks. In the 1990s, prior to the development of the current generation of transvenous ICDs with intrinsic ATP, pacemaker devices developed specifically for their ATP capabilities were implanted (often in conjunction with ICDs) to provide effective termination of VT with pacing as a preferable alternative to defibrillation or anti-arrhythmic drugs.^9,10^ Since then, initial data from the Sudden Cardiac Death in Heart Failure Trial (SCD-HeFT) established the premise that ICD shocks are associated with a substantially increased risk of death.^11^ Several analyses have also supported these findings in both trial populations and large retrospective cohorts, even after adjusting for baseline heart failure mortality.^12,13^ Several studies have observed that the increase in mortality is isolated to ICD patients receiving device shocks compared to ATP therapies alone.^14,15^ More recently, an analysis of ICD recipients in five large trials (Multicenter Automatic Defibrillator Implantation Trial II [MADIT-II], Multicenter Automated Defibrillator Implantation Trial-RISK [MADIT-RISK], Multicenter Automatic Defibrillator Implantation Trial with Cardiac Resynchronization Therapy [MADIT-CRT], Multicenter Automatic Defibrillator Implantation Trial—Reduce Inappropriate Therapy [MADIT-RIT], and Ranolazine Implantable Cardioverter-defibrillator [RAID] trial) evaluated the association of ICD therapies with subsequent mortality and found that appropriate ICD shocks were associated with the greatest risk of subsequent death, while successful ATP, failed ATP for slow VT followed by shock, and inappropriate therapies were not associated with an increased risk of death.^16^ These data demonstrate the value of preserving the ability to deliver ATP when a patient has demonstrated a successful response. Perhaps as important are the advantages in patient experience afforded by painless termination of VT with ATP. In this case, we applied a similar reasoning strategy in favor of re-implantation of a temporary transvenous RV dual-coil ICD lead over external defibrillation, a subcutaneous ICD system, or a wearable defibrillator. Epicardial lead placement could also be considered in similar scenarios if surgical management of endocarditis was indicated.

Regardless of the strategy used, it is important to maintain the ability to provide backup defibrillation in the case of failed ATP. In our case, the use of a dual-coil lead allowed programming of shock therapies using only the internal (SVC coil to RV coil) shock vector and thus avoided the need for an additional wearable defibrillator or intensive care unit-level monitoring prior to permanent device re-implantation. While most patients with a bloodstream infection and ventricular arrhythmias will require inpatient monitoring or treatment, the device configuration we have described could also provide ATP and shock therapies in the outpatient setting for those being discharged prior to permanent device re-implantation.

The technique of using a temporary external ICD system has been described in a limited amount of prior case reports in similar patients requiring ICD extraction who are at high risk for ventricular arrhythmias. Cooper et al. reported the use of an active fixation pacemaker lead to provide ATP burst therapies only in a patient with ICD infection and recurrent VT responsive to ATP.^17^ Other case reports and series have been documented in Italy (Dell’Era et al., Falasconi et al.) and Poland (Dębski et al.) with the use of dual-coil ICD leads and external ICD generators similarly to in our patient.^18–20^ These cases also highlight the ability to use ATP and ICD shocks while awaiting permanent device re-implantation, as well as overdrive pacing in patients with bradycardia-mediated ventricular arrhythmias. The limitations of the approach we describe are primarily related to the theoretical increase in the risk of re-infection with the continuous presence of endovascular hardware, as the use of a temporary device has been documented to be a risk factor for CIED infection.^3^ However, as demonstrated, in a patient with active ongoing ventricular arrhythmias, it is a safe option to balance standard-of-care treatment of the infection and safe and optimal management of monomorphic VT. This case adds to the literature describing a feasible and reliable management option for high-risk patients with CIED infections and ongoing ventricular arrhythmias. To the best of our knowledge, this case report is the first one in the literature to describe the use of a dual-coil lead with an external ICD generator in a patient being treated in the United States. Future investigations could consider broader use of this device configuration in contexts outside of CIED infection, such as critically ill hospitalized patients with concurrent ventricular arrhythmias, to provide a temporary device-based treatment option until a patient is stable and appropriate to receive a permanent ICD system.

## Conclusion

Management of recurrent ventricular arrhythmias resistant to pharmacologic therapy and responsive to ATP can be challenging in the setting of transvenous ICD infections requiring device extraction. This case demonstrates the utility of a temporary transvenous dual-coil ICD lead in combination with an externalized ICD generator to successfully detect and pace-terminate recurrent VT, thereby avoiding the need for external defibrillator therapy.
